# Dendritic cell immunometabolism - a potential therapeutic target for allergic diseases

**DOI:** 10.7150/ijms.105532

**Published:** 2025-01-01

**Authors:** Chuang Ouyang, Jiaying Huang, Gonghua Huang, Yanyan Wang

**Affiliations:** Guangdong Provincial Key Laboratory of Medical Immunology and Molecular Diagnostics, School of Medical Technology, Guangdong Medical University. Dongguan, Guangdong 523808, China.

**Keywords:** Dendritic cell, Immunometabolism, Allergic disease, T cell response, Gut microbiota, Targeted therapy

## Abstract

Allergic diseases are a group of chronic inflammatory disorders driven by abnormal immune responses. Dendritic cells (DCs) play a pivotal role in the initiation and progression of allergic diseases by modulating T cell responses. Extensive progress has been made in characterizing crucial roles of metabolic reprogramming in the regulation of immune cell functions. As the critical upstream regulators and effectors in allergic responses, the activation, migration, and function of DCs are reliant on metabolic reprogramming. In this review, we summarize the metabolic characteristics of DCs, and how the cellular microenvironment shapes DC function. We also elucidate the metabolic regulation of DC biology in the context of allergic diseases and targeted therapeutic strategies based on DC metabolism regulation. Understanding the functional alterations in DCs during allergic responses and the underlying mechanisms governing its metabolic regulation is crucial for the development of effective strategies for the prevention and treatment of allergic diseases.

## 1. Introduction

The incidence of allergic diseases has risen significantly over the past few decades, imposing a considerable burden on both patients' quality of life and healthcare systems. The pathogenesis of allergic diseases is closely linked to immune responses mediated by type 2 helper T (Th2) cells. Specifically, Th2 cells secrete cytokines, such as IL-4, IL-5, and IL-13, which promote IgE production by plasma cells, eosinophil activation, and disruption of mucosal barriers, thereby playing a central role in the pathogenesis of allergic reactions.

As the most critical and potent antigen-presenting cells (APCs), dendritic cells (DCs) recognize, capture, and process foreign antigens to peptides and present them to T cells, thus initiating and modulating adaptive immune responses. In the progression of allergic diseases, DCs regulate the differentiation and activation of Th2 cells not only by presenting antigen peptides and secreting cytokines but also by shaping the local inflammatory microenvironment through the release of inflammatory mediators and chemokines. Recent studies have shown that immunometabolism affects the maturation, antigen presentation, cytokine secretion, and function of DCs. In this review, we first provide a brief overview of the fundamental biological characteristics of DCs and their pivotal roles in allergic diseases. Next, we discuss the metabolic features of DCs and how metabolic regulators influence DC metabolic reprogramming to modulate Th2 cell differentiation and allergic diseases. We then discuss how the overall cellular microenvironment affects DC metabolism and function. Further, we review the mechanistic links between DC metabolism and common allergic diseases, along with potential therapeutic strategies by targeting DC metabolism. Finally, we highlight current research limitations. A better understanding of the relationship between DC immunometabolism and allergic responses could advance therapeutic strategies for allergic diseases.

## 2. Overview of DCs and immunometabolism

### 2.1 The heterogeneity and function of DCs

DCs, first discovered by Ralph Steinman and Zanvil Cohn in 1973, are specialized APCs found in all mammalian tissues, named for their tree-like shape^1^. As a central component of the innate immune system, DCs are capable of phagocytosing, processing, and presenting antigens, thereby initiating antigen-specific T cell activation. They serve as key regulators of T cell-mediated immunity and immune tolerance, acting as a bridge between innate and adaptive immunity^2^. Under steady-state conditions, DCs are primarily composed of three subsets: type 1 and type 2 conventional DCs (cDC1s and cDC2s, respectively) and plasmacytoid DCs (pDCs)^2^, ^3^. Additional DC subsets include epidermal Langerhans cells and monocyte-derived DCs (moDCs) that arise under inflammatory or infectious conditions^2^, ^3^. cDCs are uniquely efficient at activating naïve T cells. In both mice and humans, cDCs express CD11c and major histocompatibility complex class II (MHC-II) molecules. The development of cDC1s requiring the transcription factors IRF8, BATF3, and ID2^2^, ^3^ and cDC2s requiring IRF4^4^. Mature cDC1s express Xcr1, Clec9a, CD8α, and CD24 in lymphoid tissues and CD103 in non-lymphoid tissues, whereas cDC2s express CD172a and CD11b in lymphoid tissues and exhibit greater heterogeneity overall compared to cDC1s^5^. In contrast to cDCs, pDCs represent a distinct lineage that requires the transcription factors ZEB2 and E2-2 for development^2^, and mature pDCs express B220, Siglec-H, and bone marrow stromal antigen 2 (BST2)^6^. Beyond their developmental and phenotypic differences, DC subsets also differ functionally. cDC1s are involved in cross-presentation of antigens via MHC-I molecules to CD8^+^ T cells, activating CD8^+^ T cells to eliminate pathogens and enhance anti-tumor immune responses. cDC2s primarily present antigens on MHC-II molecules, activating CD4^+^ T cells to mediate Th2-related immune responses against parasites and allergens^2^, ^7^. Compared to cDCs, pDCs have limited antigen-presenting capacity, with their primary function being the production of type I interferons during viral infections^8^. Additionally, DCs can be categorized into immature DCs, mature DCs, and regulatory DCs based on their differentiation stages.

In terms of biological function and characteristics, DCs serve as key sentinel cells distributed throughout the body, particularly in lymphoid organs and at interfaces with the external environment, where they constantly monitor for external danger signals^9^. In the absence of inflammatory signals, the continuous environmental sensing by DCs is thought to enhance peripheral T cell tolerance, contributing to the maintenance of immune homeostasis^10^. When pattern recognition receptors (PRRs) detect pathogen-associated molecular patterns or damage-associated molecular patterns, DCs are activated or induced to mature^11^. Upon activation, DCs upregulate C-C chemokine receptor 7 (CCR7), which facilitates the migration of antigen-bearing DCs from peripheral tissues to draining lymph nodes, where they activate antigen-specific T cells^12^. In addition, PRR activation also increases the expression of MHC molecules, costimulatory molecules (CD40, CD80, CD86, et al.), and various cytokines (such as IL-12, IL-10, IL-23, TNF-α), transforming quiescent immature DCs into fully functional mature DCs capable of activating and shaping T cells^13^. Activated DCs undergo significant metabolic changes, exhibiting a metabolic profile similar to the Warburg effect found in tumor cells^14^, ^15^. Emerging evidence indicates that DCs integrate multiple environmental signals and undergo metabolic reprogramming, a process that is essential for both initiating adaptive immune responses and maintaining immune tolerance^16^, ^17^. Further understanding the links between metabolic reprogramming and the functional specialization of DCs, as well as how DC metabolism shapes T cell responses, could pave the way for developing novel therapeutic strategies to combat disease or maintain immune homeostasis. However, the precise relationship between DC metabolism and various diseases remains insufficiently elucidated to date.

### 2.2 The role of DC immunometabolism in inflammatory diseases

DCs play a pivotal role in initiating and regulating inflammatory responses^18^-^20^. They integrate metabolic signals into immune responses, making metabolic reprogramming essential not only for their activation and function but also for shaping the progression of various inflammatory diseases. In endotoxin (LPS)-driven sepsis, LPS activates TLR4, leading to a metabolic shift in DCs characterized by enhanced glycolysis and reduced oxidative phosphorylation (OXPHOS)^21^, ^22^. This shift is manifested by increased glucose consumption and lactate production in DCs, which supports the rapid production of proinflammatory cytokines, such as IL-6, IL-8, MIP-1β, and TNF-α, thereby amplifying acute systemic inflammation^21^, ^22^. Similarly, in sterile inflammation, such as the foreign body reactions induced by biomaterials, the immune metabolic signals within the biomaterial implantation microenvironment drastically influence the composition and activation status of immune cells, such as DCs and macrophages^23^. Specifically, in implant-triggered inflammatory responses, the biomaterial microenvironment undergoes glycolytic reprogramming^23^. Inhibition of glycolysis using glycolytic inhibitors, such as 2-DG, reduces the proportion of proinflammatory DCs while increasing the proportion of transitional and anti-inflammatory DCs. This shift further modulates the inflammatory response and regenerative capacity of surrounding tissues^23^. Additionally, the immunometabolism of DCs is also particularly important in allergic inflammatory responses. Tumor cells under aerobic conditions predominantly rely on aerobic glycolysis, a phenomenon known as the Warburg effect^24^. Similarly, during allergic inflammation, DCs undergo significant and highly specific metabolic changes, characterized by a general shift toward glycolysis^14^, ^24^. This metabolic reprogramming aligns with the systemic Warburg-like metabolic characteristics observed in patients with severe allergic respiratory diseases^25^. Therefore, understanding the intrinsic mechanisms underlying metabolic reprogramming in DCs could be key to unraveling the progression of severe allergic phenotypes.

### 2.3 The critical roles of DCs in allergic diseases

Classical allergic reactions include allergic rhinitis, allergic asthma, atopic dermatitis, and food allergy, which have been classified by the World Health Organization as among the most prevalent chronic diseases globally^26^. The pathophysiology of allergic diseases is mainly characterized by IgE-mediated inflammation and type 2 immune responses, in which Th2 cells play a central role by secreting cytokines such as IL-4, IL-5, and IL-13^2^, ^27^, ^28^.

As the bridge between innate and adaptive immunity, DCs are critical in the initiation and progression of allergic diseases^2^, ^27^, ^28^. Under physiological conditions, DCs maintain the balance of Th2 cell function and participate in humoral immunity to defend against extracellular pathogens. However, DC dysregulation can lead to excessive Th2 responses, which directly or indirectly trigger allergic inflammation and further develop into Th2-type allergic diseases. Increasing evidence suggests that DCs are both necessary and sufficient for initiating and sustaining Th2-type allergic responses^27^-^31^. Upon allergen exposure, DCs are activated and drive naïve CD4^+^ T cell polarization towards a Th2 phenotype through three signals: MHC-II-antigen peptide complexes, costimulatory molecules, and polarized cytokines. Simultaneously, cytokines such as thymic stromal lymphopoietin and IL-33 secreted by mucosal epithelial cells enhance OX40L expression on DCs while suppressing IL-12 production, further promoting Th2 cell differentiation and function^29^, ^30^. Phythian-Adams et al. were the first to conclusively demonstrate that DCs are crucial for initiating Th2 immune responses. In the context of *Schistosoma mansoni* infection or exposure to schistosome egg antigens, the inducible depletion of CD11c^+^ cells (encompassing all cDCs, pDCs, and certain monocytes) reduces the production of Th2 cytokines^31^. Additionally, transferring DCs from allergen (helminth)-exposed mice into naïve recipients promotes IL-4 production by CD4^+^ T cells, independent of antigen specificity of the reactive T cells^32^. This suggests that DCs exposed to allergic environments can express unique signals sufficient to drive CD4^+^ T cell differentiation toward a Th2 phenotype. In the lungs, both DCs and alveolar macrophages express high levels of CD11c. However, only the reconstitution of DCs could restore type 2 immune responses in CD11c-DTR mice treated with diphtheria toxin^33^. A single-cell RNA sequencing study further highlighted that, compared to bacterial and fungal pathogens, the uptake of helminth allergens is primarily carried out by migratory cDC2 subset, rather than other immune cell populations. These migratory cDC2s transport allergens to draining lymph nodes and induce a Th2-type immune response^34^. In addition to initiating Th2 responses, cDCs are indispensable during the effector phase of allergic reactions. Depletion of CD11c^+^ cells during intranasal allergen challenge prevents the development of airway hyperreactivity^30^, ^35^. Therefore, DCs play a central role in both initiating and maintaining allergen-driven Th2 immune responses.

### 2.4 The link between DC metabolism and allergic responses

Prior to activation by allergens or PRR ligands, DCs exhibit relatively poor immunogenicity, a process closely linked to their metabolic reprogramming^36^. Resting or immature DCs primarily generate ATP through mitochondrial OXPHOS. However, activation of Toll-like receptors (TLRs) rapidly enhances glycolysis, promotes the accumulation of succinate and the production of citrate, which is used for the synthesis of fatty acids and prostaglandins, and expands membrane structures in the endoplasmic reticulum and the Golgi apparatus for effective antigen presentation and T cell activation. This TLR-mediated “glycolytic burst” supports DC immunogenicity in allergic conditions by enhancing the synthesis and secretion of inflammatory cytokines^15^, ^36^-^38^. The use of mouse models with the mechanistic target of rapamycin (mTOR) gene depletion or rapamycin has further elucidated the role of DC metabolism in modulating allergic diseases. mTOR, a crucial regulator of cellular metabolism, affects DC homeostasis and function by promoting protein synthesis, glycolysis, mitochondrial function, and lipid synthesis^39^. Ablation of mTOR in CD11c^+^ cells results in decreased Th2-type factors such as eosinophils, IL-4, and IL-5, but increased serum IgE levels. Moreover, CD11b^+^ DCs in the lungs can shift allergic inflammation from an eosinophil-dominated Th2 response to a neutrophil-dominated Th17 response, which is associated with the production of IL-23 by DCs and an increase of fatty acid oxidation (FAO)^39^. Additionally, simultaneous treatment with rapamycin during intranasal house dust mite (HDM) administration significantly reduces the infiltration of inflammatory cells, eosinophil counts, and levels of Th2 cytokines^39^. Benito-Villalvilla and colleagues recently demonstrated that in the presence of allergen-mannan conjugates, the differentiation of monocytes is reprogrammed through metabolic reprogramming and epigenetic changes, resulting in the generation of tolerogenic DCs in allergic subjects. These tolerogenic DCs promote the development of Foxp3^+^ regulatory T (Treg) cells, reduce the production of proinflammatory cytokines TNF-α and miRNA-155, and increase the levels of anti-inflammatory miRNA-146a/b, thereby modulating the allergic disease process. During this process, the generated tolerogenic DCs shift their metabolism from the Warburg effect and lactate production to OXPHOS^40^. Itaconic acid, converted from the decarboxylation of the tricarboxylic acid (TCA) cycle intermediate *cis*-aconitate, possesses anti-inflammatory functions and emerges as a promising therapeutic target to treat allergic diseases. DCs lacking *Irg1* (immune response gene 1, encoding the enzyme *cis*-aconitate decarboxylase) exhibit enhanced ability to uptake allergens and present antigens to CD4^+^ T cells, showing more pronounced Th2-type allergic characteristics. This phenomenon might be related to the gradual activation of *Irg1* in DCs via a mitochondrial superoxide-dependent pathway in response to HDM stimulation^41^. In the context of food allergy, Sun et al. recently observed significant changes in the lipid profiles and metabolism of splenic DCs in a BALB/c mouse model of allergy induced by shrimp protein extract, highlighting notable metabolic alterations under allergen sensitization^42^.

**Figure 1:**
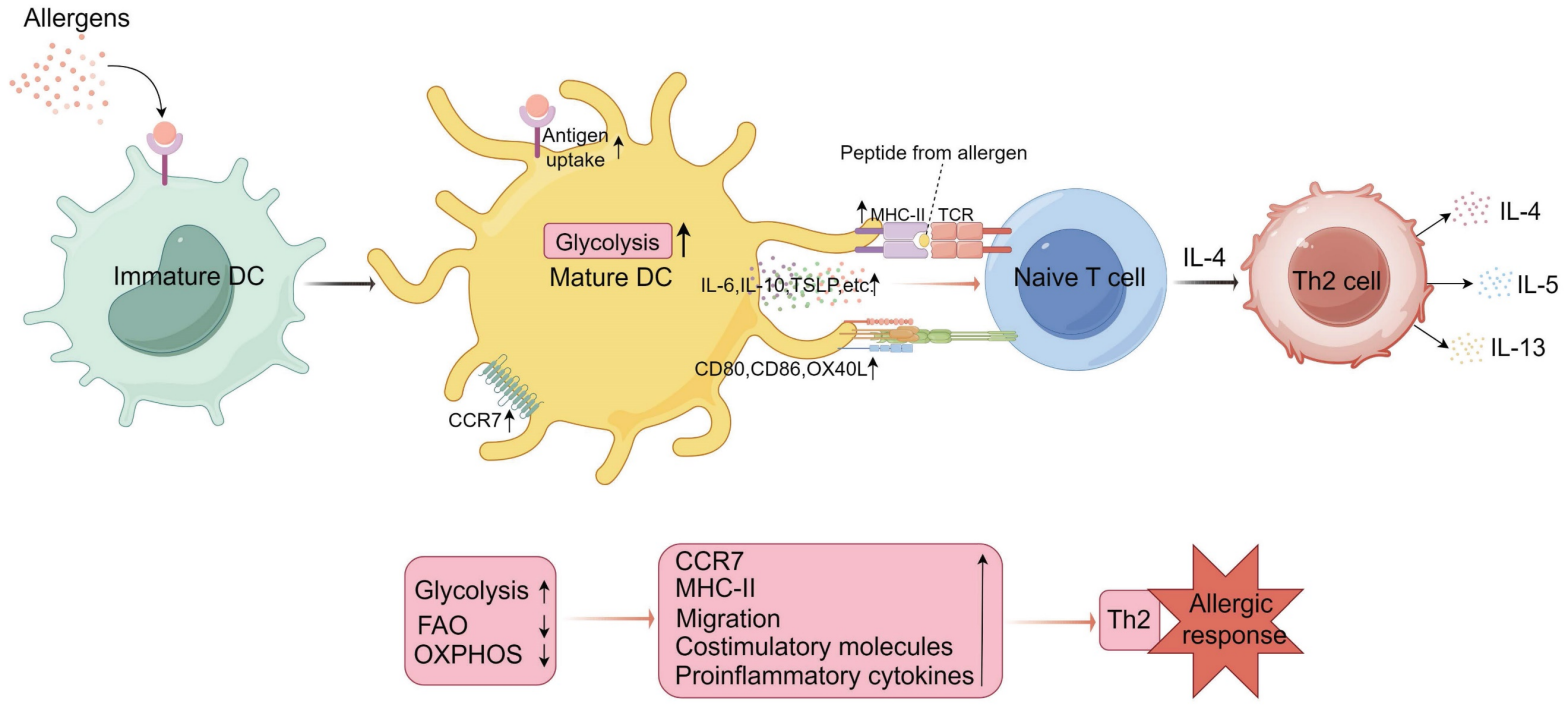
** Metabolic programming of dendritic cells (DCs) in mediating Th2 cell differentiation.** Upon uptake of external allergens, immature DCs are activated to mature and their metabolic profile is altered. Activated and mature DCs upregulate glycolytic pathways, a metabolic state that facilitates CCR7 (C-C chemokine receptor 7) oligomerization, supporting the migration of activated DCs to lymph nodes. This metabolic state also enhances the expression of major histocompatibility complex class II (MHC-II) molecules, costimulatory molecules, and proinflammatory cytokines. These factors collectively promote the activation of naïve T cells by DCs. Under the influence of IL-4, activated naïve T cells differentiate into Th2 cells, which subsequently secrete cytokines such as IL-4, IL-5, and IL-13, influencing eosinophils and B cells, thereby facilitating the onset of allergic responses. IL, interleukin; TCR, T cell receptor; FAO, fatty acid oxidation; OXPHOS, oxidative phosphorylation; TSLP, thymic stromal lymphopoietin. (By Figdraw).

Additionally, oxidative stress induced by air pollution can inhibit DC maturation under TLR stimulation, reduce IFN-γ production by T cells, decrease Th1 responses while increasing Th2 responses. Similarly, upon allergen stimulation, mucosal epithelial cells excessively produce reactive oxygen species (ROS) through activation of NADPH oxidase and NF-κB signaling pathways, leading to elevated levels of IL-1, IL-5, IL-6, IL-33, and thymic stromal lymphopoietin. Increased intracellular ROS also causes mitochondrial damage, harmful metabolite production, and DNA oxidative damage, which can lead to cell apoptosis or autophagy, thereby weakening the epithelial barrier and facilitating allergen entry into the mucosa. These metabolic microenvironment changes further impact DC function, thus exacerbating allergic symptoms^14^, ^43^-^45^. Overall, these studies confirm the strong association between DC metabolism and Th2 allergic responses, providing a theoretical basis for further research into the underlying mechanisms (**Figure 1**).

## 3. The metabolic characteristics and pathways of DCs

### 3.1 The metabolic features of DCs in resting, activated, and migratory states

**Figure 2:**
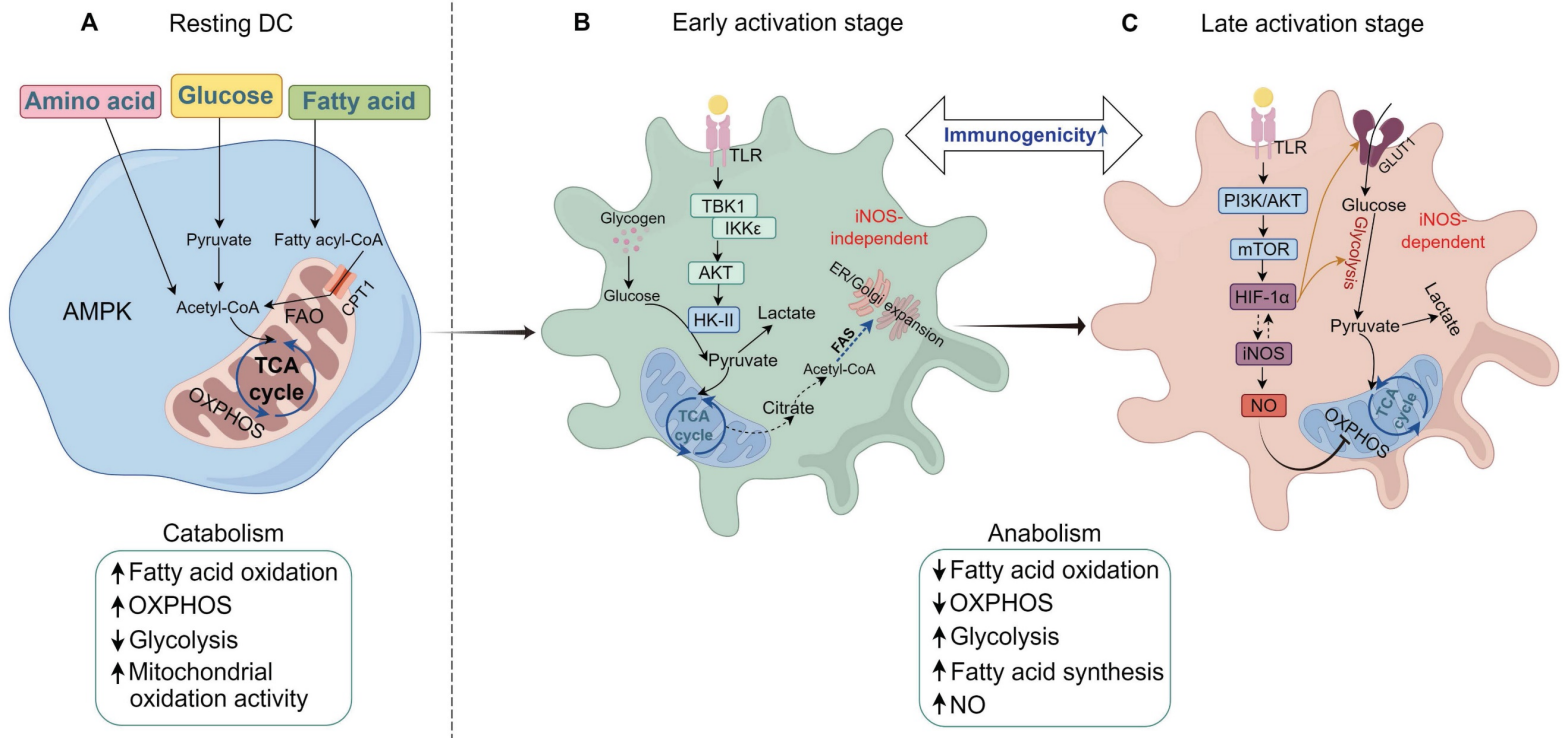
** Characteristics and mechanisms of metabolic reprogramming in DCs.** (**A**) In resting immature DCs, energy is primarily generated through catabolic pathways, such as fatty acid oxidation (FAO) and oxidative phosphorylation (OXPHOS), during which AMP-activated protein kinase (AMPK) activity is significantly upregulated. At this stage, the immunogenicity of DCs remains low. (**B**) With the uptake of allergens or TLR (Toll-like receptor) ligands, DCs are progressively activated and undergo acute metabolic reprogramming, shifting from catabolism to anabolism. At the early stage of DC activation, non-iNOS-dependent glycolysis occurs through the activation of the TBK1/IKKε pathway. Concurrently, the phosphorylation of HK-II enhances hexokinase activity, resulting in increased glycolytic levels and elevated immunogenicity of DCs. This glycolytic process also supports fatty acid synthesis (FAS), facilitating the expansion of the endoplasmic reticulum and Golgi apparatus in response to acute external stimuli. Intracellular glycogen reserves provide the necessary fuel for glycolysis during this phase. Intracellular glycogen stores provide fuel for glycolysis during this phase. (**C**) At the late stage of DC activation, iNOS-dependent glycolytic metabolism occurs. The mTOR/HIF-1α/iNOS axis in DCs is activated, further enhancing glycolysis by increasing glucose uptake and inhibiting OXPHOS. At this stage, DCs maintain a high level of immunogenicity. TCA, tricarboxylic acid cycle; CPT1, carnitine palmitoyltransferase I; GLUT1, glucose transporter 1; NO, nitric oxide; iNOS, inducible nitric oxide synthase; mTOR, mechanistic target of rapamycin; HIF-1α, hypoxia-inducible factor-1 alpha; TBK1, TANK-binding kinase 1; AKT, protein kinase B; IKKε, IκB kinase epsilon. (By Figdraw).

Current research indicates that, in the resting or inactive state, DCs predominantly engage in high levels of catabolic processes, such as OXPHOS and FAO. Resting DCs have lower energy metabolic demands and reduced immunogenicity. The catabolism of complex molecules provides substrates for the TCA cycle within mitochondria, with the breakdown of proteins and triglycerides supplying amino acids and fatty acids, respectively. These substrates are then utilized through OXPHOS to fuel ATP production^15^. Within mitochondria, the electron transport chain acquires electrons from NADH produced by the TCA cycle. NADH levels are primarily regulated by the liver kinase B1 (LKB1)-AMP-activated protein kinase (AMPK) axis. LKB1 activates AMPK and AMPK-related kinases, leading to the upregulation of catabolic pathways and mitochondrial biogenesis, while simultaneously suppressing anabolic processes to maintain this metabolic resting state^15^, ^46^. Upon activation, DCs transition their metabolic pathways towards anabolic metabolism, including glycolysis, fatty acid synthesis (FAS), and ROS production, to meet elevated energy and biosynthetic requirements^47^. Instead of relying on FAO and OXPHOS, activated DCs predominantly utilize glycolysis and lactate fermentation to meet their energy demands. Meanwhile, DCs transition from immune tolerance to immunogenicity, which corresponds with the increased levels of glycolysis that facilitate the production of proinflammatory cytokines, the expression of MHC-II and costimulatory molecules, enhanced migration, and stronger capability to activate T cells^48^, ^49^. At the early stage of activation, bone marrow-derived DCs (BMDCs) exhibit a transient increase in mitochondrial membrane potential and glycolytic metabolism, primarily driven by the TBK1-IKKε/AKT/HK-II axis. This enhanced metabolic activity predominantly supports FAS, facilitating the expansion of the endoplasmic reticulum and Golgi apparatus^37^. In contrast to the early phase of DC activation, where glycolysis occurs independently of iNOS, the later stages of DC activation rely on iNOS-dependent glycolytic metabolism (**Figure 2**). At the later stages of strong stimulation, the mTOR/hypoxia-inducible factor (HIF)-1α/iNOS axis in BMDCs is activated, which upregulates glucose transporter GLUT1 and enhances glycolytic activity by inhibiting OXPHOS through nitric oxide (NO) production. The increased glycolysis is NO-dependent and crucial for the interaction between DCs and T cells^37^, ^50^, ^51^. DC migration is also regulated by their metabolism. Guak et al. discovered that, following TLR activation, glycolysis supports the maintenance of DC shape and motility as well as the oligomerization of CCR7. These effects facilitate the efficient migration of DCs to draining lymph nodes and their subsequent activation of T cells. Additionally, during allergic inflammation, the administration of the glycolysis inhibitor 2-DG reduces the migration of DCs to the lungs under the influence of HDM^15^, ^50^. Therefore, the metabolic characteristics of DCs are intricately associated with their capacity to activate, migrate, and elicit effective T cell responses.

### 3.2 The effect of metabolic pathways in DCs on Th2 cell polarization

DCs are crucial for the induction of Th2-type allergic responses. However, the polarization signals initiated by DCs triggering these responses are not yet fully understood, especially the mechanisms by which DC metabolism influences Th2 cell polarization.

mTOR and AMPK are key signaling molecules that regulate cellular metabolism. As a downstream target of the PI3K/AKT pathway, mTOR is a major upstream activator of glycolytic reprogramming, driving the high metabolic demands of DCs after TLR activation^52^. Inhibition of mTORC1 reduces glucose consumption and lactate production, and downregulates the expression of glycolytic enzymes and glucose transporters. At the same time, it increases the extracellular acidification rate (ECAR) of BMDCs after 20 hours or more of LPS stimulation. Consistent with this, knockdown of HIF-1α, the downstream target of mTORC1, impairs BMDC maturation and late-stage glycolytic metabolism under LPS stimulation, while also reducing the production of IL-12 and TNF-α^15^, ^53^. Furthermore, DCs with a deficiency of TSC1 (a negative regulator of mTOR) exhibit increased glycolysis, mitochondrial respiration, and lipid synthesis, which promotes DC maturation^54^. However, a recent study by Tünde Fekete et al. revealed that rapamycin disrupts proinflammatory cytokine production in human moDCs by activating RIG-I-like receptor, a PRR that recognizes viral RNA. These findings indicate that mTOR exhibits both proinflammatory and anti-inflammatory effects in DCs depending on the environmental context^55^. Therefore, mTOR may be closely associated with the activation and functional phenotype of DCs. HIF-1α has a similar effect. However, its primary role is to sustain glycolysis in DCs after activation, rather than early induction^56^. Activated DCs promote autocrine IFN-I signaling through HIF-1α, which reduces OXPHOS and enhances glycolytic flux, ensuring sufficient ATP for the activation and survival of cDCs^57^. Under inflammatory conditions, the absence of HIF-1α in DCs leads to reduced glucose consumption, impaired DC maturation, and diminished ability to stimulate T cell responses^15^, ^53^. In contrast to mTOR, activation of AMPK inhibits mTOR-dependent glycolytic reprogramming while promoting catabolic pathways. AMPK shifts cellular metabolism toward energy conservation by activating the peroxisome proliferator-activated receptor-γ (PPAR-γ) coactivator-1α (PGC-1α) signaling axis, which increases mitochondrial enzyme activity and OXPHOS. AMPK can inhibit mTORC1 directly or through TSC2^54^, ^58^, ^59^. Knockdown of AMPK while stimulating TLR enhances the expression of IL-12p40 and CD86 in BMDCs, whereas AMPK activation inhibits glycolysis and the maturation process in LPS-treated DCs. Above findings indicate that AMPK acts as a negative regulator of DC activation^60^. Furthermore, specific deletion of AMPK-activating kinase LKB1 in DCs increases susceptibility to cancer and protects mice from asthma, indicating a potential anti-inflammatory role for AMPK. In addition, mice lacking LKB1 in CD11c^+^ cells exhibit a higher number of cDC2s compared to cDC1s in the thymus^46^. These phenomena suggest that AMPK is involved in DC differentiation and the induction of immune tolerance. Nieves et al. found that the deficiency of AMPK activity in myeloid DCs negatively impacts type 2 immune responses by upregulating IL-12/IL-23p40 expression^61^, suggesting that AMPK signaling may also be involved in the induction of Th2 cell differentiation by DCs. In addition, the PI3K/AKT/mTOR pathway, along with the p38, ERK1/2, and STAT3 signaling axes, is also believed to be involved in regulating the metabolic state of DCs^15^, ^62^. In a dust mite-induced Th2 asthma model, pulmonary cDC2s exhibit a pathogenic phenotype characterized by TNFR2 expression, which promotes Th2 responses. However, IFN-β treatment induces upregulation of FAO through activation of ERK2 signaling, reprogramming these pathogenic TNFR2^+^ cDC2s into tolerogenic DCs. This shift promotes the generation of pulmonary Treg cells and alleviates allergic asthma symptoms^63^.

Similarly, conditional deletion of mTOR in CD11c^+^ cells reprograms cDC2s, facilitating a shift in the allergic phenotype from a Th2 to a Th17 response^39^. Moreover, during metabolic stress, Sirtuin 1, a NAD(+)-dependent deacetylase, activates AMPK, leading to the inhibition of FAS *in vitro*. This process alters the differentiation of T cells induced by DCs both *in vitro* and *in vivo*, shifting the response from Th2/Th17 to Th1^64^, ^65^. Interestingly, Sirtuin 1 has also been shown to promote Th2 allergic immune responses by inhibiting PPAR-γ activity in DCs^64^, ^65^. We recently discovered that p38α in cDC1s affects Th2 cell differentiation during the sensitization phase of allergic asthma by regulating the MK2-c-FOS-IL-12 axis, thereby influencing disease progression^30^.

Therefore, the metabolic regulators of DCs and the metabolic pathways they control are crucial in guiding DC behavior and function. These effects also significantly affect T cell differentiation and function, and these metabolic signaling pathways are essential for the development of allergic diseases. In current research, although there is a preliminary understanding of the metabolic regulation mechanisms of DCs in Th2 cell polarization, existing studies mainly focus on the roles of individual signaling pathways, such as mTOR and AMPK. The interactions between these pathways and how they collectively regulate the overall metabolic network of DCs remain unclear. Additionally, current research primarily focuses on the overall function of DCs. However, the metabolic dependencies of different DC subsets and their specific roles in Th2 cell polarization and allergic reactions through metabolic regulation are not yet clear. Future research should further explore this area.

## 4. The influences of cellular microenvironment on DC metabolic reprogramming and function

DCs require nutrient-derived energy to perform their functions and exert effector responses. Consequently, factors in the cellular microenvironment, such as glucose, lipids, amino acids, and pH, are crucial in shaping the functional properties of DCs. It has been shown that human peripheral blood moDCs cultured in high-glucose conditions exhibit increased expression of CD86, CD83, and inflammatory cytokines IL-6 and IL-12^66^. The high-glucose environment also enhances the ability of DCs to uptake oxidized low-density lipoprotein, while upregulating ROS production and p38 expression^66^. These phenomena suggest that high levels of glucose promote DC maturation and activation. Similarly, elevated fructose levels induce DC activation by promoting the secretion of IL-1β and IL-6, and DCs exposed to fructose induce T cells to produce IFN-γ^66^, ^67^. However, unlike glucose, fructose-mediated DC activation is associated with the activation of the receptor for advanced glycation end products and does not rely on metabolic regulators^66^, ^67^. In addition to directly utilizing extracellular glucose, BMDCs and moDCs can also mobilize intracellular glycogen stores to fuel glycolysis. These glycogen stores directly participate in FAS, significantly impacting DC activation and T cell effector responses^15^, ^38^. In contrast to the effects of a high-glucose environment that may promote DC activation, a low-glucose local microenvironment more effectively supports T cell activation and immune responses during DC-T cell interactions, which may be related to the clearance of glucose by activated T cells^68^. These findings underscore the critical role of glucose in shaping DC function.

Lipid metabolism is also critical for the development and function of DCs. On one hand, lipid concentration significantly influences the immunogenicity of DCs. Hepatic DCs with high intracellular lipid content more effectively activate T cells, NK cells, and NKT cells, while those with lower lipid levels are better at promoting Treg cell-mediated immune tolerance^69^. Similarly, reducing lipid levels on BMDC membranes via high-density lipoprotein and apolipoprotein A-I induces DC tolerance and diminishes T cell responses^37^, ^70^. Additionally, inhibiting FAS in BMDCs by applying the fatty acid synthase inhibitor C75 or blocking the mitochondrial-cytosolic citrate shuttle can reduce lipid accumulation, resulting in the suppression of BMDC activation and proinflammatory functions in response to LPS stimulation^37^, ^70^. On the other hand, FAS can lead to increased lipid storage in lipid bodies within BMDCs. Lipid bodies have been shown to play a significant regulatory role in antigen cross-presentation of DCs^37^, ^71^. Additionally, the pH of the cellular microenvironment can also influence DC cross-presentation. Most proteases degrade antigens at acidic pH. Maintaining a strongly alkaline pH in cross-presentation areas can inhibit protease activity, prevent excessive antigen degradation and preserve antigenic epitopes crucial for T cell activation. This effect is associated with the recruitment of NADPH oxidase 2 to early phagosomes in DCs, which helps maintain a persistently alkaline environment within phagosomes^72^.

Beyond glucose and lipids, amino acids and vitamins in the microenvironment also play pivotal roles in DC regulation. Amino acids, the essential nutrients for immune cells, not only serve as the fundamental building blocks of proteins, but also act as alternative sources for glucose and fatty acids. Reducing the supraphysiological concentrations of amino acids commonly found in standard culture media to levels comparable to those in human plasma has been shown to enhance the efficiency of moDC differentiation^73^, ^74^. Under similar amino acid concentrations, moDCs cultured with plasma from patients with advanced liver cirrhosis—where various amino acid imbalances are present—exhibit impaired maturation, IL-12 secretion, and migration following LPS stimulation. This imbalance disrupts mitochondrial TCA activity and OXPHOS, leading to decreased intracellular ATP levels in immature DCs^73^, ^74^. Further research demonstrated that LPS treatment increases the uptake of branched-chain amino acids, such as isoleucine, leucine, and valine, in moDCs. Notably, valine deficiency suppresses moDC maturation, downregulates CD83 expression, and impairs their ability to induce T cell proliferation, possibly due to compromised mTOR signaling^74^, ^75^. LPS treatment also increases the uptake of glutamate and cysteine by moDCs and leads to an increase in ROS levels. Inhibiting the activity of the cystine/glutamate antiporter reduces glutathione synthesis without affecting moDC antigen uptake or maturation, but impairs the ability of DCs to present antigens to both CD4^+^ and CD8^+^ T cells, given that glutathione is known for its antioxidant properties^73^, ^76^. This phenomenon may be related to the role of DCs in maintaining redox homeostasis in the DC-T cell interaction microenvironment through glutathione export^73^, ^76^. Glutamine, a precursor in glutamate synthesis, has recently been identified as an intercellular metabolic checkpoint that governs the crosstalk between tumors and cDC1s, enabling cDC1s to activate CD8^+^ T cells^77^. cDC1s and tumor cells compete for glutamine in the tumor microenvironment via the transporter protein SLC38A2 to modulate anti-tumor immunity, and glutamine is the key amino acid promoting the antigen-presenting function of cDC1s^77^. Lastly, vitamins, as essential micronutrients, have been found to regulate DC function by influencing their metabolic reprogramming. Treatment with vitamin D3 induces the generation of tolerogenic DCs. In addition, vitamin D3-induced tolerogenic DCs upregulate OXPHOS and FAO pathways, while enhancing glycolytic capacity^78^. This form of metabolic reprogramming likely underpins their immunosuppressive phenotype.

In summary, nutrients and homeostasis in the microenvironment profoundly influence the immune functions (e.g., immunogenicity, immune tolerance) and metabolic states of DCs. However, our understanding of how nutrients in distinct tissue microenvironments mediate immune responses and their precise mechanisms remains limited. Furthermore, most studies on these microenvironments have been conducted *in vitro*, which does not accurately capture the complexity of the metabolic microenvironments *in vivo*.

## 5. The regulation of allergic diseases by modulating DC metabolism

### 5.1 Allergic asthma

Allergic asthma is a disease characterized by chronic airway inflammation, with clinical symptoms such as recurrent wheezing, shortness of breath, chest tightness, and coughing, primarily triggered by environmental allergens like HDM and pollen^79^. Antigens inhaled through the respiratory tract are captured by DCs within the airway lumen, whereupon antigen-activated DCs migrate to the draining lymph nodes in the lungs to present antigens to naïve CD4^+^ T cells. During this process, DCs upregulate costimulatory molecules and cytokines (e.g., OX40L, CCL17), which induce the differentiation and activation of Th2 cells, leading to the production of IL-4 and further exacerbating inflammatory response in allergic asthma^29^, ^30^, ^79^. Recent studies have shown that DCs play a crucial role in regulating Th2 response, and inhibiting DC activity can downregulate Th2 response, making DCs a potential target for treating allergic asthma^30^, ^80^.

FAO is not only one of the most critical metabolic pathways in the body but also a core regulatory factor in various physiological and pathological processes, including allergic diseases, obesity-related metabolic disorders, and cancer. Importantly, FAO plays a significant role in maintaining and establishing the phenotype and function of immune cells^14^, ^15^, ^81^. FAO primarily occurs in mitochondria, where it undergoes activation, translocation, β-oxidation, and is eventually fully oxidized via the TCA cycle to produce CO_2_ and H_2_O, releasing energy. CPT I and CPT II are crucial enzymes catalyzing this process. Their activity is regulated by multiple signaling pathways, including AMPK, PPAR, STAT3, and PGC-1 pathways. Therefore, these pathways are critical not only for regulating FAO, but also for affecting immune cell function^17^. Resting DCs generate energy through catabolic pathways such as OXPHOS and FAO, which is regulated by AMPK/PGC-1α. Compared to immunogenic DCs, tolerogenic DCs exhibit more active catabolic features related to FAO and OXPHOS^15^, ^60^, ^82^. These findings suggest that FAO might impact on DC function. *In vitro* experiments demonstrated that FLT3L stimulation induces the differentiation of DC progenitors into cDC1s, cDC2s, and pDCs, and treatment with etomoxir, a CPT I inhibitor, promotes the differentiation of cDC2s over cDC1s without affecting pDCs, suggesting that FAO may contribute to the development of specific DC subsets^46^, ^83^. This process is closely linked to the AMPK pathway, and AMPK activation favors mitochondrial FAO over cytosolic FAS^46^, ^83^. Stimulation of GM-CSF-derived BMDCs by TLRs results in reduced AMPK activation and FAO. Similarly, targeting *Prkaa1*, which encodes the AMPKα1 subunit, with shRNAs leads to increased expression of CD86 and IL-12p40 in LPS-induced BMDCs^60^. Moreover, targeted silencing of the key enzyme in FAO, CPT1A, inhibits FAO but restores the ability of DCs to activate CD8^+^ T cell proliferation. This effect has been demonstrated to be associated with the regulation of FAO in DCs by the Wnt5a-β-catenin signaling pathway through the PPAR-γ-CPT1A axis^84^. Collectively, FAO may promote a tolerogenic phenotype in DCs. Recently, Kaushik Sen et al. also discovered that inhibition of FAO can alter the tolerogenic phenotype of primary human moDCs^85^. Therefore, FAO metabolism impacts the biological functions and phenotypes of DCs, and these changes are crucial for DC-mediated Th2-type allergic responses. The role of FAO in modulating DC function to influence the Th2 phenotype has been validated through metabolic pathways such as PPAR and AMPK. For instance, *L. gasseri* has been shown to inhibit airway inflammation by activating PPAR-γ in DCs^86^. This finding is consistent with the work of Hammad et al., who demonstrated that activation of PPAR-γ promotes DC tolerance and impairs DC maturation, thereby reducing eosinophilic airway inflammation in mouse models of asthma^87^. Conversely, through inhibition of PPAR-γ activity in DCs, sirtuin 1 drives DC maturation towards a Th2-promoting phenotype and exacerbates Th2 allergic airway inflammation^64^. Thus, activation of PPAR-γ in DCs enhances FAO and promotes a tolerogenic phenotype in DCs, thereby mitigating allergen-induced airway inflammation. As above mentioned, AMPK is associated with DC-induced Th2 cell differentiation. AMPK promotes FAO, mitochondrial OXPHOS, and other forms of catabolism while acting as a negative regulator of DC activation^54^, ^58^-^61^. Although PGC-1 and STAT3 are known to regulate FAO, there is still limited research on how they influence the immune metabolism and function of DCs in the context of allergic diseases. Overall, FAO influences the induction and maintenance of allergic responses by modulating DC function, providing insights for the development of new therapeutic strategies. Future research should further explore the functional significance and mechanistic basis of FAO in DC biology.

### 5.2 The gut microbiota-DC metabolic axis and food allergy

Increasing evidence suggests that the occurrence of food allergy is associated with abnormal composition and metabolic changes of the gut microbiota. The gut microbiota play a critical role in maintaining immune cell homeostasis and the balance of the immune system, while dysregulation of gut microbiota can promote the development of food allergy^88^, ^89^. DC metabolism, as a novel immunomodulatory perspective, plays a significant role in maintaining intestinal homeostasis through its metabolic interactions with gut microbiota. Food allergy is an immune disorder primarily characterized by a Th2 cell immune response, manifesting as intolerance to specific foods. The onset of food allergy is largely driven by immune signals resulting from disrupted tissue homeostasis, rather than the intrinsic allergenicity of the food itself. This immune response leads to the activation of the innate immune system, which, in conjunction with food allergens, promotes a specific Th2 immune response^90^, ^91^. Intestinal DCs, predominantly located in the lamina propria and mesenteric lymph nodes, capture antigens and activate Th2 cells to produce cytokines, which in turn induce B cells to produce antigen-specific IgE, triggering food allergy^90^, ^91^.

The gut and its microbiota have co-evolved, with gut microbiota playing a crucial role in maintaining the intestinal barrier. The diversity and abundance of specific bacterial groups have been shown to be closely linked to the development of food allergy^88^, ^92^, and intestinal DCs are essential for maintaining this intestinal homeostasis. In *Zbtb46*-DTR mice, the absence of pre-DCs disrupts oral tolerance to ovalbumin antigens, while the reduction of CD11c^+^ DCs leads to spontaneous autoimmune diseases and inflammatory bowel disease^93^, ^94^. However, excessive DC production of proinflammatory cytokines IL-12p40 and IL-23 disrupts gut microbiota homeostasis, thereby promoting intestinal inflammation^93^, ^94^. Therefore, the inflammatory state of DCs can disrupt gut homeostasis. In contrast, gut microbiota-derived metabolites such as short-chain fatty acids (SCFAs), bile acids, and amino acids possess immunoregulatory functions that significantly impact DC function and metabolic state. A recent work by Wu et al. uncovered that depletion of normal gut microbiota and antibiotic-induced disruption of bile acid homeostasis enhance retinoic acid (RA) signaling in mucosal DCs^95^. Importantly, RA signaling within DCs is necessary for the production of food allergen-specific IgE and IgG1 antibodies^95^. The activation of RA signaling promotes the upregulation of type I interferon signaling, RA signaling, OX40L, and PD-L2 in DCs, which may drive the differentiation of CD4^+^ T cells towards a Th2 phenotype^95^. Thus, the interaction between gut microbiota, their metabolites, and DCs likely plays a crucial role in the pathogenesis of food allergy. Further research has shown that the regulation of RA signaling by gut bacteria is specific to intestinal DCs^95^, ^96^. BMDCs deficient in RARα, the most critical transcriptional regulator in RA signaling, exhibit a marked inhibition of the differentiation of naïve CD4^+^ T cells into IL-4^+^ CD4^+^ T cells^95^, ^96^. In contrast, the RARα-specific agonist, tamibarotene, enhances Th2 cell differentiation^95^, ^96^. These findings suggest that RARα may contribute to the modulation of allergic responses by guiding the differentiation of Th2 cells. Among the most abundant metabolic products of gut microbiota, SCFAs, including butyrate, acetate, and propionate, have been implicated in the development of food allergy^89^. Additionally, SCFAs function as effective immunomodulators, regulating DC activity and thereby influencing allergic symptoms^89^. Their effects are primarily mediated through the inhibition of histone deacetylases and the activation of G protein-coupled receptors^89^. Butyrate, the most abundant SCFAs in the gut, along with propionate, can inhibit the expression of proinflammatory cytokines in DCs by suppressing histone deacetylase activity, thereby promoting the differentiation of intestinal Treg cells to maintain gut homeostasis^97^. Additionally, both butyrate and propionate can inhibit the expression of maturation markers (such as CD83) and the production of proinflammatory chemokines and cytokines in human moDCs induced by LPS, demonstrating potent anti-inflammatory effects^98^, ^99^. Consistent with this, butyrate also promotes an anti-inflammatory phenotype in CD103^+^ DCs in the colon through GPR109A, leading to increased Treg cells and IL-10 production^98^, ^99^. Tan et al. observed that a high-fiber diet can remodel gut microbiota and increase the levels of acetate and butyrate in the intestine. These SCFAs enhance the activity of retinaldehyde dehydrogenase in CD103^+^ DCs via GPR43 and GPR109A, thereby improving oral tolerance and preventing food allergy^100^. Thus, SCFAs exert anti-inflammatory effects on DCs, thereby effectively mitigating symptoms of food allergy.

In addition to SCFAs, other gut microbiota-derived metabolites such as bile acids and the toxic lipid 12,13-diHOME also play crucial roles in regulating DC function and maintaining intestinal homeostasis. Unlike SCFAs, which can alleviate allergy symptoms, treatment of human DCs with 12,13-diHOME results in reduced IL-10 secretion and decreased anti-inflammatory Treg cells^101^, suggesting that 12,13-diHOME may promote allergic inflammation by inducing DC dysfunction. This process is mediated by the interaction between 12,13-diHOME and PPAR-γ in DCs^101^. Unlike 12,13-diHOME, the secondary bile acid derivative 3β-hydroxydeoxycholic acid (isoDCA) attenuates the immunostimulatory properties of DCs, thereby enhancing their ability to induce Treg cell differentiation in the gut. This immunomodulatory effect is linked to the inhibition of the nuclear farnesoid X receptor activity in DCs^102^. Additionally, gut microbiota and their metabolites direct the unique transcriptional, epigenetic, and basal metabolic states of cDCs by controlling the sustained production of IFN-I signals from pDCs, allowing them to rapidly respond to pathogens and initiate adaptive immune responses^103^.

As noted above, gut microbiota and their metabolites, in coordination with DCs, work together to maintain the homeostasis of the gut and the overall immune system. Future research that delves deeper into how gut microbiota influence the metabolism and epigenetics of DCs may shed light on the biological functions and immune regulatory mechanisms of DCs, providing a new immunological basis for the treatment of food allergy and gastrointestinal diseases.

## 6. Targeted therapeutic strategies based on DC metabolism regulation

Currently, allergen immunotherapy (AIT) remains the primary treatment for allergic diseases. AIT involves gradually increasing the dose of allergen extracts to repeatedly expose patients to the allergens, thereby enhancing their tolerance and reducing allergic symptoms^104^. However, long-term AIT can lead to immune dysregulation and other side effects, and its efficacy may be limited in specific diseases^104^. Therefore, the development of new therapeutic approaches may enhance both the specificity and effectiveness of treatment.

DCs serve as important mediators of allergic responses, and alterations of their metabolic states can significantly influence their effector functions. Thus, targeting metabolic pathways and related signaling networks, or modulating the metabolic state of DCs, may represent a viable strategy for developing novel therapeutic approaches. mTOR and AMPK are the most central energy metabolism sensors and regulators in DCs as well as important links in the regulation of allergic responses by DCs. Pharmacologically targeting these pathways may offer potential therapeutic benefits for allergic conditions. In an allergic asthma model, treatment with rapamycin concurrent with HDM exposure alleviates airway inflammation^39^, ^105^. However, administering rapamycin after HDM exposure fails to reverse the condition and instead exacerbates allergic symptoms, suggesting that the efficacy of rapamycin may be environment-dependent^39^, ^105^. AMPK generally acts as a negative regulator and has anti-inflammatory effects in DCs. Oral administration of metformin (an AMPK activator) reduces airway inflammation and decreases pulmonary eosinophil counts in ovalbumin-induced obese allergic mice^46^, ^60^, ^106^. Further, activation of PPAR-γ in DCs has been shown to enhance DC tolerance and impair their migration, thereby mitigating asthma symptoms^87^. The selective agonist rosiglitazone significantly reduces airway inflammation and effectively maintains pulmonary immune homeostasis following PPAR-γ activation^87^. Consequently, both metformin and rosiglitazone may emerge as viable therapeutic strategies. Recent studies indicate that the cAMP/protein kinase A signaling pathway in DCs plays a critical role in regulating Th2 states and allergic asthma. cAMP, a second messenger closely associated with cell proliferation, differentiation, and metabolic activities, regulates cellular functions through the activation of protein kinase A^107^, ^108^. Elevated levels of cAMP in DCs can reduce the release of proinflammatory cytokines, such as TNF-α, and increase the production of the anti-inflammatory cytokine IL-10, exhibiting promising therapeutic effects in Th2 cell-medicated allergic diseases^107^, ^108^. Treatment of mouse DCs *ex vivo* with the cAMP analogue 8-(4-Chlorophenylthio)adenosine 3',5'-cyclic AMP results in reduced IL-4 secretion and GATA3 expression, and alleviates inflammation, although it increases IL-17A secretion^107^, ^108^. This provides a new direction for targeted therapies in allergic diseases. In terms of targeting DC functional phenotypes, dexamethasone and vitamin D3 have been shown to induce tolerance in DCs and thereby reduce inflammation^109^-^111^. Specifically, vitamin D3-treated DCs promote the expansion of Treg cells by inducing ILT3 expression and inhibit NF-κB activation through binding to vitamin D receptors (VDR) on DCs, thus fostering the generation of tolerant DCs^109^-^111^. Tolerant DCs generated using vitamin D3, NF-κB inhibitors, or VDR agonists have been utilized as DC vaccines for disease treatment^109^-^111^. In the future, integrating nanotechnology could enhance the targeting and efficacy of DC vaccines, potentially offering new strategies for treating allergic diseases.

The primary methods for delivering these inhibitors or activators still rely on conventional drug administration techniques, including oral intake, injections, or topical applications. However, these approaches are often cause side effects and lack the precision to specifically target immune cells. Biomaterial-based strategies, such as drug delivery technologies involving liposomes, nanoparticles, microspheres, exosomes have emerged as effective alternatives. These technologies enable precise drug delivery to specific organs or cells, enhancing therapeutic targeting while minimizing off-target effects^112^, ^113^. By reducing systemic side effects, these advanced strategies allow comprehensive control over the spatial, temporal, and dosage distribution of drugs, thereby improving patient outcomes^112^, ^113^. For respiratory diseases, nanometer-scale drug delivery systems have been developed to encapsulate drugs in surface-modified nanoparticles or microparticles for inhalation-based delivery. This approach allows these particles to cross the biological barriers of the lungs, enabling targeted delivery to affected regions^113^, ^114^. Further surface modifications enable nanoparticle recognition of specific receptors in diseased regions, enhancing targeted delivery and local drug accumulation^113^, ^114^. In allergic asthma, nanoparticle drug delivery systems can encapsulate agents capable of modulating the metabolic state of DCs, enabling precise targeting of DC-enriched areas. This targeted approach has the potential to modulate DC activity and improve asthma symptoms. Similar systems could be applied to other allergic diseases, focusing on modulating DC metabolism to alleviate allergic conditions. Recently, Mangal and colleagues developed a strategy for sustained local delivery of metabolites into cells using alpha-ketoglutarate (αKG) and diol-based polymeric-microparticles (pαKG MPs). These particles modulate the metabolism of immune cells by binding to DCs and altering their metabolic profile, including reducing glycolysis and mitochondrial respiration^115^. These metabolic changes affect the expression of MHC-II and CD86 on DCs, and influence the frequencies of Treg cells as well as other helper T cell subsets^115^. This unique strategy for the sustained delivery of key metabolites into cells provides a promising foundation for immunometabolism-based therapies. It demonstrates the feasibility of targeting DC metabolism through biomaterial-based drug delivery systems, paving the way for novel immunotherapeutic strategies.

Altering dietary components is another viable and effective strategy for preventing allergic diseases^116^. Gut microbiota and their metabolites can influence DC metabolism and function, and are closely associated with allergic conditions. Therefore, inducing the production of specific SCFAs through dietary fiber intake to alleviate symptoms of food allergy or asthma is a feasible approach^116^, ^117^. Probiotics also play a beneficial role in the prevention of allergic diseases. For example, the probiotic *Lactobacillus rhamnosus* GG alleviates asthma symptoms in mice by reducing the expression of surface molecules on DCs and increasing the induction of Treg cells by CD103^+^ DCs in the mesenteric lymph nodes^118^, ^119^. Other probiotics, such as *Lactobacillus reuteri* and *Bifidobacterium breve*, can significantly reduce the secretion of Th2 inflammatory cytokines while enhancing the diversity of the gut microbiota^118^, ^119^. However, the specific effects of these probiotics on DCs are not yet fully understood. Additionally, reprogramming the metabolic state of DCs by modulating nutrient availability may represent an effective strategy for controlling inflammation. For instance, altering glucose or lipid levels in the microenvironment could have significant effects. A recent study has shown that dietary supplementation with specific triacylglycerols or ceramides modulates the immune function of DCs, thereby alleviating Th2 immune responses induced by shrimp allergen proteins^42^. Altogether, modifying the diet or administering specific types of probiotics can both prevent and alleviate allergy symptoms.

To date, there are still few targeted drugs available for clinical treatment, with most remaining in the animal model or cell experiment stages. Modulating the metabolic states or pathways of DCs holds the potential for precise regulation of allergic responses and may also reduce the side effects associated with traditional treatments. Immune therapies based on DC metabolism have demonstrated substantial application potential. Future strategies could include developing new therapeutic approaches based on DC metabolism, such as using CRISPR-Cas9 technology to knockout or knockin specific metabolism-related genes to modulate DC function and alleviate or suppress allergic diseases. Alternatively, small interfering RNA could be delivered to targeted sites via nanoparticle-based systems to suppress the expression of DC metabolic genes associated with allergies, thereby controlling the onset and progression of allergic diseases at their source. Another promising direction is the development of advanced biomaterials and drug delivery systems for precise targeting of DC metabolism. This strategy holds the potential to accurately regulate allergic responses while significantly minimizing the side effects associated with conventional therapies, or identifying more specific metabolic biomarkers for highly precise and targeted treatments.

## 7. Conclusions and perspectives

In recent years, significant progress has been made in understanding the regulation of DC metabolism. However, research on DC immunometabolism remains in its early stages, with many fundamental questions still unresolved. In this review, we explore the specific mechanisms through which DC metabolism regulates allergic diseases. However, studies on the detailed connections between DC metabolism and allergic conditions are still limited. Current research reveals that metabolic reprogramming plays a crucial role in determining DC functions and in the pathogenesis of allergic diseases. Notably, alterations in glucose and lipid metabolism pathways, as well as changes in the overall metabolic microenvironment, significantly impact DC maturation, antigen presentation, and immunoregulatory functions. These metabolic changes directly or indirectly influence T cell activation and differentiation, thereby playing a significant role in the development of allergic diseases. Metabolic regulators such as AMPK and mTOR are not only critical pathways for DC function but also pivotal links between DCs and allergic diseases. The research on DC metabolism so far predominantly employs *in vitro* models using human moDCs or mouse BMDCs. These studies typically alter individual genes or specific metabolites under conditions of ample nutrients and oxygen. However, such *in vitro* culture models overlook the impact of *in situ* microenvironment on the metabolic complexity of DCs. Therefore, the development of culture systems that more closely mimic the *in vivo* microenvironment could provide a more robust foundation for studying DC metabolism in the future. Moreover, the intercellular metabolic crosstalk between different DC subsets and between DCs and other immune or non-immune cells in various microenvironments remains poorly understood. Unraveling these mechanisms could offer new insights for developing more effective therapeutic strategies. Looking ahead, a comprehensive approach integrating genomics, metabolomics, transcriptomics, and proteomics could systematically elucidate the role of DC metabolism in allergic diseases and other immune-related disorders, thereby providing a foundation for personalized treatments and the development of new therapeutic targets. Furthermore, the development of well-designed biomaterial-based local drug delivery systems to precisely target DCs, modulate their metabolic state and improve allergic responses represent an exciting yet underexplored area. Future research could prioritize optimizing the biocompatibility, targeting specificity, and release mechanisms of these delivery systems, offering innovative and precise therapeutic strategies for allergic and other immune-related diseases.
